# Evolution of COVID-19 in the State of São Paulo: Analysis of Incidence, Mortality and Lethality from 2020 to 2023

**DOI:** 10.3390/epidemiologia6010006

**Published:** 2025-02-06

**Authors:** Lybio Jose Martire Junior, Gabrielle do Amaral Virginio Pereira, Matheus Paiva Emidio Cavalcanti, Yasmin Esther Barreto, Hugo Macedo, Fernando Augusto Marinho dos Santos Figueira, Romildo Luiz Monteiro Andrade, Luiz Carlos de Abreu

**Affiliations:** 1 Postgraduate Program in Health Sciences, Centro Universitário da Faculdade de Medicina do ABC, Santo André 09060-650, SP, Brazil; lybiojunior@gmail.com (L.J.M.J.); hugomacedojr@hotmail.com (H.M.J.); 2 COVID-19 Observatory Brazil and Ireland, School of Medicine, University of Limerick, V94 T9PX Limerick, Ireland; gabrielleamaral@usp.br (G.d.A.V.P.); mpaivaemidio@gmail.com (M.P.E.C.); yasminebarreto@usp.br (Y.E.B.); fernando.figueira@imip.org.br (F.A.M.d.S.F.); 3 Department of Medicine, Itajubá School of Medicine, Itajubá 37502-138, MG, Brazil; 4 Postgraduate Program in Medical Sciences, Faculty of Medicine, University of São Paulo, São Paulo 01246-903, SP, Brazil; 5 Laboratory of Design of Studies and Scientific Writing, Federal University of Espirito Santo, Vitória 29075-910, ES, Brazil

**Keywords:** COVID-19, coronavirus, time series studies, epidemiology

## Abstract

Introduction: COVID-19 is a respiratory disease caused by the SARS-CoV-2 virus, which belongs to the coronavirus family. SARS-CoV-2 is related to other viruses that cause severe acute respiratory syndrome. The emergence of cases of pneumonia of unknown origin triggered the largest viral pandemic in modern times, presenting major challenges to global public health. Objective: To analyze the evolution of the COVID-19 pandemic in the state of São Paulo from 2020 to 2023, focusing on trends in incidence, mortality, and lethality. Methods: Ecological study of time series of incidence, mortality and lethality by COVID-19 in the state of São Paulo using Prais-Winsten regression considering the Weekly Percentage Change (WPC) and probability values (p), considering a significance level of 95% (95% CI). To ensure the reliability of the entered data, double-blind typing was performed by different researchers in the same database extracted from the 2024 Ministry of Health Coronavirus dashboard. Results: From February 2020 and the end of December 2023, 6,763,310 accumulated cases and 182,254 deaths were recorded. Stationary trends were observed for the year 2022, with a reduction in incidence and mortality in the year 2023. However, the epidemiological variable lethality showed a stationary trend. Conclusion: The analysis of the trends in incidence, mortality, and lethality revealed variable dynamics over time, with emphasis on the significant reduction of these indicators in 2023.

## 1. Introduction

In December 2019, cases of pneumonia of unknown origin were described in the city of Wuhan, China. Weeks later, Chinese authorities identified a novel coronavirus as the cause of the disease [1]. The International Committee on Taxonomy of Viruses named the virus SARS-CoV-2 [2], and the World Health Organization (WHO) designated the disease COVID-19 [3].

With the progressive increase in confirmed cases, on 30 January 2020, the WHO declared the viral epidemic a public health emergency of international concern [1]. Called COVID-19, the viral disease was characterized by symptoms of dyspnea, fever, cough, fatigue, and muscle pain [4,5], with the possibility of worsening to Severe Acute Respiratory Syndrome (SARS) and death [6]. Aggravated by viral proliferation, COVID-19 has shown a high prevalence rate due to high transmissibility and high lethality among those infected [7]. As of 30 June 2024, approximately 776 million cases of COVID-19 had been recorded worldwide, resulting in 7.1 million deaths.

In terms of fatalities, the Americas were the most affected region, totaling 3,023,053 deaths [8]. These numbers highlight the severity of the pandemic and the continued need for surveillance and adoption of control measures. The high mortality rates across the globe underscored the importance of implementing effective public health strategies and vaccination programs to attenuate the impacts of COVID-19.

One of the main challenges in combating the pandemic was the rise of new variants of SARS-CoV-2, considering that the accumulation of mutations resulting from viral replication is a natural phenomenon. Although most of the mutations have no noticeable impact, some of them have given rise to new variants with greater infectivity and virulence [6,9].

The high mutability of SARS-CoV-2 has come to require continuous updating of treatments and booster doses of vaccines, as mutations alter the effectiveness of the vaccines developed. The development and continuous updating of newly developed vaccines have become necessary to contain the damage caused by the pandemic, both in the economic sphere and in the daily social activities [10,11]. Several non-pharmacological strategies have been proposed to try to contain the transmissibility of SARS-CoV-2, with emphasis on hand hygiene and maintaining social distancing, however, they were not enough to prevent the spread of the agent on a pandemic scale, aggravating the syndemic behavior of COVID-19 around the world [10,11].

In Brazil, since the beginning of the pandemic in January 2020 until mid-July 2024, 38,840,012 cases were registered with the Ministry of Health, of which more than 712 thousand resulted in death [12,13]. The mortality rate of 339.08 per 100,000 inhabitants [14] reveals the severity of the challenges faced by the health system, highlighting the severity of the complications caused by COVID-19. In addition, the incidence rate of 18,482.30 per 100,000 inhabitants [12] highlights the spread of the virus throughout the country, impacting urban and rural communities, large centers and even more remote regions.

The Southeast Brazilian region reported a total of 15,511,185 confirmed cases and 343,520 deaths. The State of São Paulo (SP) alone had around 6 million cases and more than 183 thousand deaths, demonstrating the severity of the health scenario. As the epicenter of the pandemic in the country, SP faced the challenges of exhaustion of hospital capacity, distribution of medical resources, in addition to efforts to contain the virus’s spread.

The high incidence of cases and deaths, combined with the state’s population density, high mobility rates, and the complexity of public health management, underscores the critical importance of studying the pandemic’s impact. SP as Brazil’s most densely populated state, presents a unique case for examining the evolution of COVID-19. This temporal series study examined the progression of the COVID-19 pandemic in the state from February 2020 to December 2023, focusing on trends in incidence, mortality, and lethality.

## 2. Materials and Methods

### 2.1. Outline of the Study

This is a population-based ecological study of the time series of incidence and lethality rates of COVID-19 that occurred in the State of SP based on reported data extracted from the Brazilian Ministry of Health CORONAVIRUS PANEL for the year 2024, accessed through the link: https://covid.saude.gov.br, extracted on 31 January 2024 [15]. The information excluded personal identifiers and included confirmed COVID-19 cases and deaths recorded from February 2020 to December 2023 [15].

### 2.2. Criterion of Inclusion and Exclusion and Collect of Data

The inclusion criteria for the cases considered included deaths of individuals with a laboratory diagnosis of COVID-19, cases reported in the CORONAVIRUS PANEL system based on the International Classification of Diseases, 10th edition (ICD-10), specifically as “U07.1 COVID-19, virus identified” or “U07.2 COVID-19, virus not identified”, or COVID-19 “U02.2”, virus not identified [15].

To ensure data accuracy, cases and deaths according to the date of notification were classified independently by two different researchers, whose results are present in the research results section. The original data are kept by the main author of this study in case they are needed for future analysis or verification.

### 2.3. Data Analysis

The data were tabulated to calculate the relative frequency, by dividing the number of confirmed cases and deaths from COVID-19 in the month and/or year by the total period evaluated and multiplying by 100, expressed as a percentage (%).

The incidence and mortality rates for COVID-19 per 100,000 inhabitants were calculated. For both indicators, monthly measurements of cases (48 months) and deaths (48 months) were used. To calculate the monthly rates, the intercensal estimates carried out by the Fundação Sistema Estadual de Análise de Dados (SEADE) for the period between 2019 and 2023 were considered, and the population data were considered on the reference date of the collection [16]. The night of 31 July to 1 August. Next, population projections were made for the calendar months of the years 2020 to 2023, with the aim of ensuring greater reliability for the analyses [12].

The incidence and mortality rates were standardized for 100,000 inhabitants and the Lethality Rate, expressed as a percentage, demonstrating the proportion of people who died among those diagnosed with COVID-19. This approach expresses the impact of COVID-19 on a standardized scale, facilitating comparison between different populations and periods. The mathematical Equations (1)–(3) indicate the calculations performed:

*DPC* = (10^β^ − 1) × 100% (1)

(*CI*95%) = (10^*βMAX*^ − 1) × 100% (2)

(*CI*95%) = (10^*βMIN*^ − 1) × 100% (3)

In calculating incidence and mortality, the population was obtained from the “Population Projection of the Federation Units by Sex and Age Group: 2000–2030” for 2020 (46,289,333 inhabitants), 2021 (46,649,132 inhabitants), 2022 (46,997,428 inhabitants) and 2023 (47,333,328) [12]. In our study, we sought to analyze how COVID-19 indicators change in 4 segments:

1- 2020 (from February to December),
2- 2021 (from January to December),
3- 2022 (from January to December) and
4- 2023 (from January to December).

The approach made it possible to observe the evolution of events in each specific time interval, favoring the accurate trend analysis according to the recommendations of Antunes and Cardoso [17]. From then on, the Prais-Winsten regression model was used to consider the influence of first-order autocorrelation in the analysis of time series data. The data modeling process included the transformation of the rates (dependent variable = Y value) into a base 10 logarithmic function, and the results of the logarithmic rates (β) allowed the estimation of the Weekly Percentage Change (WPC) and its respective confidence intervals with 95% significance. The probability (p) and DVP values were calculated using Equations (1)–(3), where β is the slope of the linear regression, the indices ul represent the upper limit and ll the lower limit of the confidence level [17,18]. Allowing the categorization of incidence, mortality and lethality trends as increasing (*p*-value < 0.05 and positive beta), decreasing (*p*-value < 0.05 and negative beta) or stable or stationary (*p*-value ≥ 0.05) [17].

Statistical analyses were performed using STATA 14.0 software College Station, TX, USA, 2013. As stated in an opinion dated 21 January 2021, by the Research Ethics Committee of the MultiVIX Faculty in Cachoeiro do Itapemirim, ES, Brazil (02.213.188/0002-62), this work does not require approval from the Research Ethics Committee.

## 3. Results

The State of SP has a population of 44,411,238 inhabitants and a density of 178.92 inhabitants per km^2^. It has a Human Development Index (HDI) of 0.806, which reflects the standard of living of the population at the time of this study [13,16].

During the study period, 6,763,310 reported cases and 182,254 deaths were recorded, represented in Table 1 with the respective incidences and monthly relative frequencies of cases and deaths. The first two cases were recorded in February 2020, representing less than 0.001% of cases, while in the month of the first death in March 2020, 136 deaths were found (0.07%).

The monthly analysis of the first year showed that July and August had the highest incidence of cases with 260,924 (3.85%) and 262,038 (3.87%) respectively. Regarding mortality, July was marked by 8234 deaths, representing 4.51% of deaths for the entire period. In 2021, it was observed that June had the highest incidence, with 455,305 (6.73%) cases recorded, and April led the number of deaths, with 21,539 fatalities.

April 2021 stands out as the month with the highest relative frequency (11.81%) of deaths in a single month. In 2022, February reported the highest number of cases and deaths, with 371,347 (5.49%) and 6678 (3.66%) registered respectively. In 2023, January had the highest number of cases and deaths, with 97,174 (1.43%) and 1064 (0.58%) recorded, respectively.

**Figure 1:**
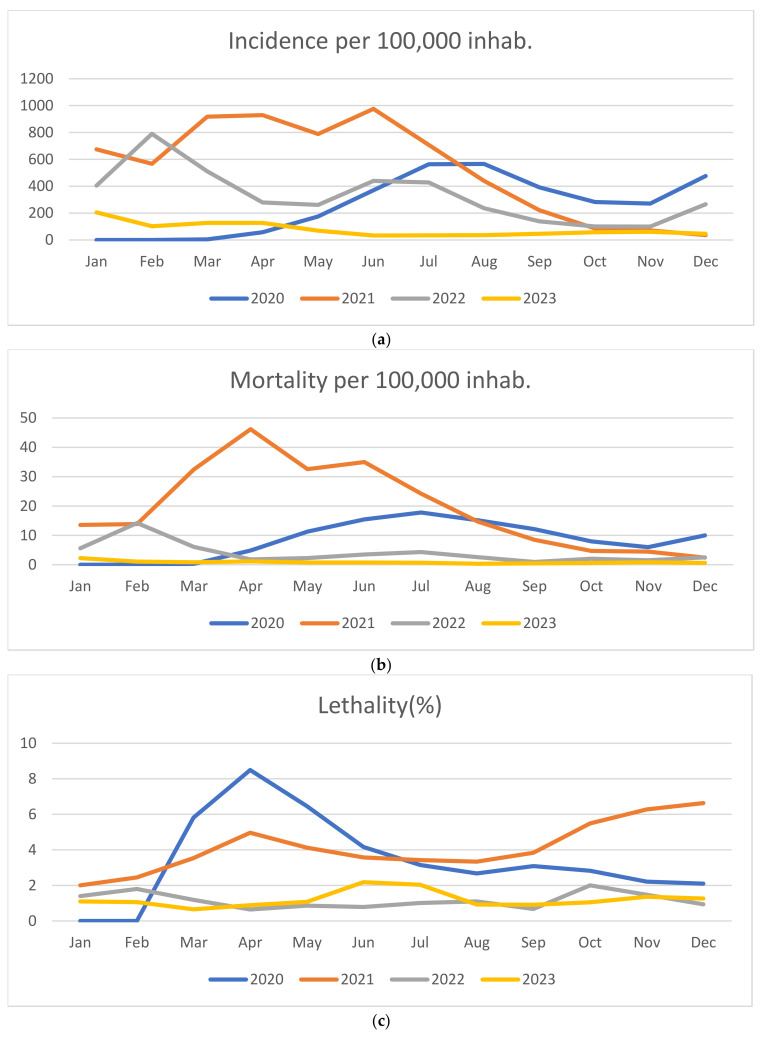
Incidence, rates of mortality and Lethality from the COVID-19 in the state of SP from January 2020 to December 2023. Temporal analyses were illustrated as described as follows: (**a**) incidence, (**b**) mortality, and (**c**) lethality of COVID-19 cases.

The highest incidence rate was observed during 2021 with peak values recorded from March (917.15 per 100,000 inhabitants) to July (976.02 per 100,000 inhabitants). High numbers were also noted in August 2020 (566.08 per 100,000 inhabitants) until the end of the year (476.66 per 100,000 inhabitants), while the lowest incidences rates occurred in 2023, with emphasis in June, recorded at 33.39 per 100,000 inhabitants. (Figure 1a).

Regarding mortality rates, the year 2021 presents the highest values, particularly from February (13.84 per 100,000 inhabitants) to August (14.66 per 100,000 inhabitants). Similarly, from August (15.15 per 100,000 inhabitants) to December (9.98 per 100,000 inhabitants) of 2020 mortality rates remained higher compared to other years. Differently, 2023 remained stable and represents the lowest rates throughout the analyzed period, with August reporting the lowest mortality rate observed during the four years (0.32 per 100,000 inhabitants), as illustrated in Figure 1b.

Regarding lethality, the graph illustrates a different pattern in relation to the other observed rates, with 2020 showing the highest peaks. Lethality rates were highest in March (5.81%) and June (4.16%), followed by an increase from July 2021 (3.42%) to December (6.63%).

The lethality rates were at their minimum between 2022 and 2023, with the lowest values in 2023 occurring in January (1.09%), March (0.65%), August (0.91%), October (1.05%), and November (1.35%). In 2022, the lowest rates were recorded in April (0.64%), July (1.01%), September (0.67%), and December (0.93%) (Figure 1c)

The COVID-19 pandemic showed distinct patterns of incidence, mortality and lethality trends (Table 2) providing a comprehensive assessment of how the pandemic progressed during the analyzed period in SP until the end of 2023. The monthly distribution of COVID-19 cases and deaths in the state totaled approximately 6 million cases and 182,000 deaths. The first deaths were recorded in March 2020, with a total of 136 deaths that month. In 2020, the highest number of deaths occurred in August and July. In 2021, June recorded the highest number of deaths, followed by April, which also had the highest relative frequency. In 2022, February saw the highest number of cases and deaths, while in 2023, January reported the highest figures for both.

The incidence showed an increasing trend for 2020 (WPC = 25.33%, *p* < 0.05) with a decrease in 2021 (WPC = −6.64%, *p* < 0.05), remaining stationary in 2022 (WPC = −1.96, *p* > 0.05) and a new decrease in 2023 (WPC = −2.51%, *p* < 0.05). For mortality, the trend remained increasing (WPC = 10.57%, *p* < 0.05) in 2020, decreasing for 2021 (WPC = −4.84%, *p* < 0.05); remaining stationary in 2022 (WPC = −1.90%, *p* > 0.05), and decreasing in 2023 (WPC = −1.98%, *p* < 0.05). Lethality had a decreasing trend in 2020 (WPC = −2.99%, *p* < 0.05), while in 2021 it was increasing (WPC = 2.44%, *p* < 0.05), being stationary in 2022 (WPC = −0.02%, *p* > 0.05) until 2023 (WPC = 0.55%, *p* > 0.05). Despite the mostly stationary trends observed in 2022, there is a decreasing behavior in incidence (WPC = −2.51%, *p* < 0.05) and mortality (WPC = −1.98%, *p* < 0.05). In 2023, however, this behavior is not observed for lethality, which presented a stationary trend (WPC = 0.55%, *p* > 0.05).

## 4. Discussion

The trajectory of the COVID-19 pandemic in the state of SP was marked by intense dynamics, with the number of deaths showing considerable variations throughout the analyzed period. The epidemic curve, with its peaks and valleys, reflected the complexity of interactions between the SARS-CoV-2 virus and society. These dynamics were influenced by various factors including the emergence of new viral variants [19,20], the implementation of control measures [21,22] and the demographic and socioeconomic characteristics of the populations affected [23,24].

Throughout the period analyzed, the highest incidence and mortality rates were recorded in 2021. Conversely, the lowest rates were observed in 2023, which can be considered the year when epidemic behavior significantly subsided, with incidence rates reaching their lowest values compared to previous years. Regarding lethality, 2020 stands out as the year with the highest rates, differing from the trends observed for incidence and mortality specific to COVID-19.

Older adults and individuals with comorbidities, such as hypertension, diabetes and cardiovascular diseases, were disproportionately vulnerable to worsening COVID-19 and deaths arising from the illness. Those groups presented a bigger risk of severe complications due to the compromise of their immune systems and the presence of pre-existing conditions that make recovery difficult [25,26]. Furthermore, social inequalities exacerbated the impact of the pandemic. People with less access to health care and living in precarious conditions face additional challenges, such as a lack of adequate medical resources and the inability to follow effective prevention measures, such as social distancing and proper hygiene. These disparities exacerbated health inequalities, making certain groups’ populations yet more vulnerable to the effects from the COVID-19 [23,24].

Consistent with the findings of this study, which revealed a decrease in the incidence rate of COVID-19 in the state of São Paulo in 2023, an additional ecological analysis highlighted that the federative units with the highest incidence rates between 2020 and 2021 were Amapá, Rio Grande do Sul, Rondônia, and Roraima. These states, located in the North and Northeast of Brazil, face significant challenges due to structural deficiencies in their health systems. In contrast, the state of São Paulo which reported a lower incidence rate, benefits from a more robust infrastructure within the Unified Health System (SUS) [27,28]

Overall, Brazil faces profound inequalities and inequities in the distribution of goods, services, and wealth, resulting from a historical accumulation unequally divided across generations. In the field of health, these inequalities are evident in variations in access to technological advancements and differences in opportunities for exposure to health determinants, disease, and mortality. As a result, the number of COVID-19 cases and related deaths increased rapidly in urban peripheries and gradually expanded to inland areas, intensifying regional and socioeconomic disparities in health [29,30,31].

The reduction in the incidence rate in the State of São Paulo suggests that mitigation and control strategies have been effective, contributing to a significant decrease in the spread of the disease compared to previous years. Similar trends were observed in other Brazilian regions and highlight the importance of continuing vaccination and epidemiological surveillance efforts to maintain control over the pandemic [32,33,34,35].

Similar to the trends in incidence, the lowest mortality rates in the state of São Paulo were observed in the final year of analysis, 2023. The start of vaccination against COVID-19 in Brazil provoked significant changes in the profiles of hospitalizations and Deaths associates the disease. An increased risk of hospitalization and mortality was observed among younger individuals who did not receive the vaccine, while the mortality rate among vaccinated elderly individuals was substantially reduced. In addition, there was an overall decrease in both hospitalization and death rates, highlighting the effectiveness of vaccination campaigns in protecting vulnerable populations and mitigating the severe impacts of the pandemic [36].

In agreement with the findings of Sobczak and Pawliczak, 2022 [37], a crucial factor in reducing COVID-19 mortality is an adequate health system combined with an accurate restriction policy. The reduction of mortality and Lethality by COVID-19 in the state of SP in 2023 can be attributed to a combination of strict preventive measures and an effective vaccination campaign.

As seen in several European countries that implemented strict restrictions and promoted awareness of the importance of hygiene, SP authorities also adopted measures such as mandatory use of masks, social distancing and campaigns of vaccination in mass. These actions collaborated to control the dissemination of the virus and protect the vulnerable population. In addition, the ready response of the government and the increased hospital capacity contributed to a decrease in hospitalizations and deaths [38,39,40].

However, regarding the incidence of COVID-19, globally, the incidence rate in the third year of the pandemic remained high [41]. The persistence of a high incidence rate can be attributed to several factors, including the continuous circulation of new variants of virus, inequalities in access to the vaccines, resistance to adherence to prevention measures and variations in public health policies between countries [42].

The vaccination evidenced to be the most effective measure to reduce the mortality related the COVID-19. It significantly reduced mortality rates among patients. In some countries, as Bulgaria, Latvia, Lithuania and Romania, the number of people fully vaccinated was positively correlated with the mortality rate. In contrast, in Hungary, Romania and Slovakia, the increase in the number of vaccine doses and boosters was also associated with an increase in COVID-19 mortality [37].

A study analyzed the relationship between the incidence rate of COVID-19 in the population of different countries and the types of vaccines used against SARS-CoV-2, considering demographic and immunobiological factors. In the Asia Eastern, the highest incidence of COVID-19 was observed in South Korea, what used predominantly messenger RNA vaccines, while the smallest incidence proportion was found in China, which used mainly inactivated virus vaccines [42].

In Europe, countries with a predominance of mRNA vaccines, such as France and Portugal, had an 18% to 45% higher incidence rate of COVID-19 than the United Kingdom, which had a balanced mix of vaccine types and superior testing coverage. This suggests that the number of COVID-19 cases depends not only on testing and vaccination coverage but also on the types of vaccines used. Countries that predominantly used vector and inactivated virus vaccines showed a significantly lower incidence rate than those that predominantly used mRNA vaccines [42].

In Brazil, nine types of vaccines have been used to combat the coronavirus. The most widely used vaccines in adults were ChAdOx1-S/nCoV-19 [recombinant] (AstraZeneca), CoronaVac (Butantan), Bnt162b2 (BioNTech/Pfizer) and Ad.26.COV2.S (Janssen Pharma) [43]. The effectiveness and the scope of the vaccines against new variants were sufficient to significantly reduce the incidence of severe acute respiratory syndrome and decrease the number of deaths and hospitalizations associated with SARS-CoV-2 [43].

Vaccination against COVID-19 in Brazilian states presents heterogeneous coverage, reflecting the country’s socioeconomic and structural disparities. Evidence revealed that states with a lower HDI, limited primary care coverage and fewer public health facilities have lower rates of primary and booster vaccination coverage [44,45].

Spatial and temporal analysis of vaccination reveals that the North and Northeast regions have lower vaccination rates compared to the Southeast and South, which concentrate most of the vaccines administered [46,47]. Furthermore, the pandemic has exacerbated existing inequalities, with indigenous and quilombola populations having the lowest vaccination rates [46,47].

However, despite of continuous efforts in vaccination campaigns and improvement of vaccine formulations, ideal prevention against virus transmission has not yet been achieved [48]. This challenge is markedly evident in the context of new variants, which present mutations capable of reducing the effectiveness of existing vaccines, highlighting the need for continuous adaptations and updates in immunizers to effectively face the evolution of the virus [48,49].

The findings of this study reveal significant variations over the analyzed period, highlighting peaks and valleys in the COVID-19 epidemic curve. These fluctuations reflect the complex interaction between the virus SARS-CoV-2, society, and the emergence of new variants. The data indicate that the pandemic in São Paulo was marked by different phases, with timelines of high transmission and mortality, followed by periods of relative stabilization. The evolution of incidence, mortality and lethality rates over time highlights the importance of continuous epidemiological surveillance strategies, vaccination and effective communication with the population to face future challenges and mitigate the impacts of new variants.

This study has some limitations: firstly, the use of secondary data extracted from the DATASUS database may lead to issues related to data quality and integrity. Another limitation is related to the generalization of the results, since the analysis was restricted to the population of SP, it is not possible to extend the conclusions to other regions of the country or to Brazil as a whole. Furthermore, external factors, such as changes in the population health or behavioral policies may influence the trends observed throughout the period analyzed, which could impact the interpretation of the results.

Despite these limitations, this study has strong points and demonstrates its significant importance. The use of a time series method provides a robust approach to examine the temporal variations of health indicators over the period, which allows identifying possible changes in trends over time, providing important information for understanding the evolution of COVID-19. In addition, the use of data coming from DATASUS allows a comprehensive and representative analysis of the epidemiological situation of COVID-19 in the state of SP. Understanding the trends and factors associated with the pandemic is crucial to guiding effective interventions of health public.

Therefore, this study contributes to the scientific knowledge about the epidemiology of COVID-19 in SP and offers essential information for the formulation of health policies and programs aimed at preventing and controlling disease, adapting to the specific needs of the population and developing improvements in response to new variants of the virus.

## 5. Conclusions

The analysis of incidence, mortality, and lethality trends revealed variable dynamics over time, with a notable reduction in these indicators in 2023. However, the persistence of high global incidence rates and the emergence of new variants highlight the continued need for epidemiological surveillance and the development of effective public health strategies. This study provides valuable insights into the temporal trends of COVID-19 in SP, contributing to the broader understanding of the pandemic’s progression. The findings offer critical information to guide the formulation of public health measures, for managing and responding to future public health crises.

## Figures and Tables

**Table 1:** Distribution from the frequency absolute and relative of cases and Deaths put COVID-19 by month and year in São Paulo state, Brazil, 2020–2023.

Year	Month	Cases		Deaths	
		No.	%	No.	%
2020	Jan	0	0	0	0
	Feb	2	<0.01	0	0
	Sea	2337	0.03	136	0.07
	Apr	26,359	0.38	2239	1.22
	May	81,000	1.19	5240	2.87
	June	171,682	2.53	7148	3.92
	Jul	260,924	3.85	8234	4.51
	Aug	262,038	3.87	7017	3.85
	Set	181,286	2.68	5608	3.07
	Out	130,499	1.92	3689	2.02
	Nov	125,526	1.85	2784	1.52
	Ten	220,644	3.26	4622	2.53
2021	Jan	315.071	4.65	6317	3.46
	Feb	264,260	3.90	6459	3.54
	Sea	428.221	6.33	15,159	8.31
	Apr	433,860	6.41	21,539	11.81
	May	368,334	5.44	15,183	8.33
	June	455.305	6.73	16,307	8.94
	Jul	330,520	4.88	11,315	6.20
	Aug	204,816	3.02	6840	3.75
	Set	103,448	1.52	3974	2.18
	Out	39,945	0.59	2192	1.20
	Nov	33,028	0.48	2075	1.13
	Ten	17.003	0.25	1128	0.61
2022	Jan	189,998	2.80	2649	1.45
	Feb	371,347	5.49	6678	3.66
	Sea	240,240	3.55	2842	1.55
	Apr	131,652	1.94	849	0.46
	May	122,594	1.81	1061	0.58
	June	206,553	3.05	1623	0.89
	Jul	200,954	2.97	2037	1.11
	Aug	111.100	1.64	1215	0.66
	Set	65,566	0.96	445	0.24
	Out	47,437	0.70	950	0.52
	Nov	46,970	0.69	695	0.38
	Ten	124,814	1.84	1162	0.63
2023	Jan	97,174	1.43	1064	0.58
	Feb	48,658	0.71	518	0.28
	Sea	60.381	0.89	395	0.21
	Apr	59,912	0.88	528	0.28
	May	32,610	0.48	351	0.19
	June	15,807	0.23	345	0.18
	Jul	16,095	0.23	327	0.17
	Aug	16,855	0.24	155	0.08
	Set	22,069	0.32	200	0.10
	Out	27,559	0.40	290	0.15
	Nov	28,912	0.42	392	0.21
	Ten	21,945	0.32	278	0.15
	Total	6,763,310	100.00	182,254	100.00

**Table 2:** Prais–Winsten regression estimates and Weekly percentage change (WPC) of incidence, mortality and Lethality of COVID-19 node state of They are Paul, Brazil, of January of 2020 the December of 2023.

Indicators	Year	Linear Regression				
		Beta	WPC	(IC 95%)	*p*	Trend
Incidence	2020	0.0980708	25.33	7.19:46.54	0.006	Growing
	2021	−0.029841	−6.64	−9.55:−3.64	<0.001	Descending
	2022	−0.008616	−1.96	−4.21:0.33	0.092	Stationary
	2023	−0.01105	−2.51	−4.43:−0.56	0.013	Descending
Mortality	2020	0.0436532	10.57	0.56:21.59	0.039	Growing
	2021	−2.15 × 10^−2^	−4.84	−9.18:−0.29	0.038	Descending
	2022	−8.33 × 10^−3^	−1.90	−4.59:0.87	0.172	Stationary
	2023	−8.67 × 10^−3^	−1.98	−3.21:−0.73	0.003	Descending
Lethality	2020	−0.013166	−2.99	−4.04:−1.92	<0.001	Descending
	2021	0.0104584	2.44	1.15:3.74	<0.001	Growing
	2022	−7.07 × 10^−5^	−0.02	−1.34:1.32	0.98	Stationary
	2023	0.002399	0.55	−0.76:1.88	0.402	Stationary

## Data Availability

The data presented in this study are available in https://covid.saude.gov.br, (Accessed on 31 January 2024). These data were derived from the following resources available in the public domain: CORONAVIRUS PANEL.
